# Human large-scale cooperation as a product of competition between cultural groups

**DOI:** 10.1038/s41467-020-14416-8

**Published:** 2020-02-04

**Authors:** Carla Handley, Sarah Mathew

**Affiliations:** 1 School of Human Evolution and Social Change, Arizona State University, P.O. Box 872402, Tempe, AZ 85287 USA; 2 Institute of Human Origins, Arizona State University, Tempe, AZ 85287 USA

**Keywords:** Anthropology, Cultural evolution, Culture, Decision making

## Abstract

A fundamental puzzle of human evolution is how we evolved to cooperate with genetically unrelated strangers in transient interactions. Group-level selection on culturally differentiated populations is one proposed explanation. We evaluate a central untested prediction of Cultural Group Selection theory, by assessing whether readiness to cooperate between individuals from different groups corresponds to the degree of cultural similarity between those groups. We documented the normative beliefs and cooperative dispositions of 759 individuals spanning nine clans nested within four pastoral ethnic groups of Kenya—the Turkana, Samburu, Rendille and Borana. We find that cooperation between groups is predicted by how culturally similar they are, suggesting that norms of cooperation in these societies have evolved under the influence of group-level selection on cultural variation. Such selection acting over human evolutionary history may explain why we cooperate readily with unrelated and unfamiliar individuals, and why humans’ unprecedented cooperative flexibility is nevertheless culturally parochial.

## Abstract (web summary)

The authors here show that readiness to cooperate between individuals from different groups corresponds to the degree of cultural similarity between those groups. This is consistent with the theory of Cultural Group Selection as an explanation for the rise of human large-scale cooperation.

## Introduction

Humans cooperate with genetically unrelated individuals in transient interactions, a striking departure from patterns of cooperation in other vertebrate societies. The main evolutionary theories for why animals cooperate do not adequately explain this unusual mode of cooperation. An emerging but controversial idea is that our species’ extreme reliance on culture facilitated such cooperation by paving the way for group-level selection to act on culturally differentiated populations^1–8^. Theorists have claimed that this process referred to as Cultural Group Selection (CGS) is possible because cultural variation is structured in populations to a greater degree than genetic variation^1,2,9,10^. The premise is that while migration steadily erodes genetic variation between groups, cultural variation between groups is preserved as migrants may adopt the cultural practices of their new group. Competition between culturally differentiated populations then leads to the evolution of social norms that benefit the cultural group. Such norms would require people to cooperate with culturally similar individuals even in transient interactions.

Although widely discussed, CGS theory has not been rigorously tested^11^. Supporting evidence is mainly indirect coming from historical and ethnographic examples that are consistent with CGS^12–14^, from arguments that the necessary conditions for CGS to operate are present in humans^3,15^, and from demonstrations of group beneficial properties of human social institutions^16–23^. Consequently, scholars are divided about how important CGS is^24–35^.

We test a key untested prediction of CGS relating the scale of cooperation to the population structure of cultural variation. Humans live in nested and overlapping social structures such as residential communities, clans, ethnic groups, and nations. This means that there are a variety of social scales at which cooperation could potentially occur. The strength of CGS at a particular social scale will be proportional to the magnitude of between-group cultural variation at that scale^15^. CGS at a particular social scale should lead to the proliferation of norms that benefit that social scale. This means that high cultural differentiation between groups of a particular scale (e.g., between clans) should be associated with parochial norms that promote cooperation with ingroup individuals (e.g., norms to help clan members) relative to cooperation with outgroup individuals (e.g., norms to help non-clan members). Cultural *F*_ST_, which is the proportion of the total variation in cultural traits that lies between populations, is a critical statistic for how strong group-level selection will be relative to individual-level selection^15,36–38^. Thus, we should see a negative association between cultural *F*_ST_ and cooperation between populations.

To illustrate the logic underlying this prediction, suppose a novel norm, X, arises within a population. Within-group social learning processes will cause the norm to spread to a population of individuals who are socially learning from each other. We refer to this culturally similar group of people as population X. Suppose norm X forbids individuals to steal livestock of people who adhere to norm X. Contrast this to a norm, Y, which restricts stealing livestock exclusively from relatives. Norm Y will also spread among people socially learning from each other, who we refer to as population Y. Norm Y does not produce the same benefits for its adherents as norm X does, as it permits stealing from adherents of norm Y. Therefore, compared to population X, population Y will decline, via reduced demographic growth, emigration to other cultural populations, or adoption of the cultural practices of successful populations. Over time, populations will tend to have norms such as X, rather than norms such as Y, generating the correspondence between the scale of cultural differentiation and the scale of cooperation that we examine here. The process thus is a “group-structured” form of cultural selection. A particular individual from population Y cannot on their own begin practicing norm X. By doing so they would violate the local norm and lower their payoffs. To get the benefits of being an adherent of norm X, they would need to emigrate to population X, or a majority of people in population Y must synchronously switch to practicing norm X.

CGS does not have to favor altruistic forms of cooperative behavior for the predicted correspondence between the social scale of cooperation and cultural variation to be observed. If CGS influences norms governing cooperation, it is in the interest of individuals to comply with these norms as deviating will lead to social disapproval and sanctions. For instance, among the Turkana, warriors who display cowardice during cattle raids are beaten and fined by their age mates, and may be less likely to receive help when they are in need^39,40^. Here we take such norm enforcement as given and examine the content of cooperative norms, particularly what they specify about who its adherents are obligated to help.

We conducted the study among subsistence pastoralists in northern Kenya drawn from four ethnolinguistic groups: the Borana, Rendille, Samburu and Turkana. They are socially organized along patrilineal descent-based exogamous clan and moiety structures (Supplementary Figs. 1–4). The Turkana also have territorial subdivisions, which are geographic distinctions indicating grazing area rights (Supplementary Fig. 4). Women adopt the social identities of their husbands if they are married in accordance with local custom, which involves the payment of bride price; otherwise they retain their father’s social identity. These populations are well suited to test CGS because there is ongoing competition for resources. Individuals from these communities subsist by herding cattle, camel, sheep and goat occupying semi-arid savanna environs adjacent to one another. Migrating periodically during dry seasons in order to access pastures and water for their livestock leads multiple communities to vie for the same grazing areas and watering sites. It is possible for communities to agree to share access to grazing areas and water sites and refrain from stealing each other’s livestock. Alternately, conflicts over resources may precipitate periods of hostility characterized by mutual cattle theft between communities, as well as large-scale armed lethal raids on other communities. During these raids the successful side appropriates their opponents’ livestock and may displace them from preferred grazing lands or water sites. As natural fertility populations, their demographic growth is strongly influenced by access to limiting resources.

**Fig. 1:**
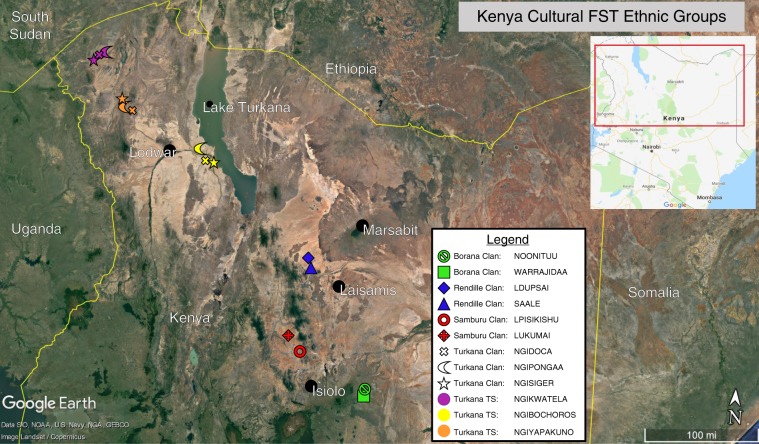
Data collection sites. GPS locations of the clans and territorial sections in the sample superimposed on Google Earth map of northern Kenya. TS, territorial section.

We sampled 793 individuals spanning 9 clans, and 3 Turkana territorial sections (Table 1 and Fig. 1). We assessed cultural differentiation based on 49 social norms pertinent to pastoral livelihood, and calculated cultural *F*_ST_ values between pairs of groups. We assessed cooperation rates using 16 vignette scenarios in which the main character is in a position to affect the well-being of the target character. Because geographic proximity could also influence cooperation either directly by facilitating social interactions, or indirectly by maintaining cultural homegeneity, we calculated geographic distance between groups based on subjects’ GPS coordinates at the time of the study. We find that the rate of cooperation is strongly associated with cultural *F*_ST_ values between pairs of groups, and is not strongly associated with geographic distance. Specifically, low cultural *F*_ST_ between pairs of groups predicts high levels of cooperation across the groups. This correspondance between the population structure of cultural variation and the social scale of cooperation suggests that competition between cultural groups has shaped cooperation norms in these populations.

**Table 1:** Study populations and sample size.

Ethnic group	Language genus^a^	Spoken language	Social organization	Approx. population in Kenya^b^	Clans sampled (sample size)	Gender of sample (% female)
Borana	Lowland East Cushitic	Southern Oromo	2 exogamous moieties 17 clans	160,000^c^	Noonituu (52) Warrajidaa (50)	49%
Rendille	Lowland East Cushitic	Kirendille	2 phratries 9 exogamous clans	60,000	Ldupsai (66)^d^ Saale (70)	66%
Samburu	Nilotic	Northern Maa	2 phratries 8 exogamous clans	250,000	Lpisikishu (52) Lukumai (51)	50%
Turkana	Nilotic	Kiturkana	18 territorial sections (TS), 24 exogamous clans cross-cutting the TS	1,000,000	Ngikwatela TS: Ngisiger (50), Ngipongaa (50), Ngidoca (50) Ngiyapakuno TS: Ngisiger (51), Ngipongaa (48), Ngidoca (50) Ngibochoros TS: Ngisiger (51), Ngipongaa (52), Ngidoca (50)	68%

^a^Numbers reported here are based on estimates provided by *World Atlas of Languages (WALS) Online.*

^b^Numbers reported here were obtained from the 2014 census report of the *Kenya National Bureau of Statistics.*

^c^Borana extend into Ethiopia so their total population exceeds the numbers living in Kenya.

^d^15 Ldupsai and 19 Saale participants were only administered the cooperation questionnaires as described in the Methods.

## Results

### Cultural differentiation among the study groups

**Fig. 2:**
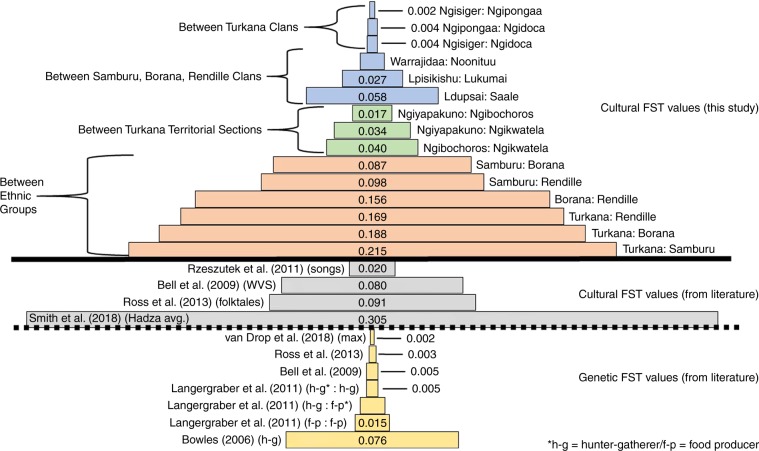
Cultural differentiation between groups. Top portion shows cultural *F*_ST_ values averaged across 49 traits between pairs of clans within ethnic groups (blue), pairs of territorial sections within Turkana (green) and pairs of ethnic groups (brown) in our study. Estimates are based on a sample of 759 individuals as shown in Table 1. Bottom portion shows cultural *F*_ST_ values (gray) and genetic *F*_ST_ values (yellow) from the literature. Source data are provided as a Source Data file.

Cultural *F*_ST_ values are sizable, especially between ethnolinguistic groups, indicating that there is scope for CGS to occur. The top portion of Fig. 2 displays the *F*_ST_ values averaged across the 49 cultural traits for each of the pairs of social groupings: pairs of clans within each of the ethnolinguistic groups, pairs of territorial sections of the Turkana, and pairs of ethnolinguistic groups. See the Source Data file for pairwise *F*_ST_ values for each individual trait. The *F*_ST_ values between clans and between territorial sections range from 0.002 to 0.058. The *F*_ST_ values between ethnolinguistic groups are considerably higher, ranging from 0.087 to 0.215. This means that up to a fifth of the variation in traits can lie between groups, even in fairly large groups spanning several hundred thousand individuals. For large groups such as the populations studied here, *F*_ST_ approximately equals the coefficient of relatedness ***r***, indicating that assortment is possible among cooperators, creating the conditions for cooperation to evolve ^41^.

### Cultural differentiation levels compared to previous studies

The cultural *F*_ST_ values between ethnolinguistic groups in our study are within the range of cultural *F*_ST_ values documented in the literature to date^15,36–38,42^, and an order of magnitude higher than relevant genetic *F*_ST_ estimates^15,36,43–45^. Bell et al.^15^ calculated cultural *F*_ST_ values between neighboring countries using data from the *World Value Survey*, which measures individual attitudes and beliefs in a wide range of contexts. Rzeszutek et al.^37^ examined 421 traditional group songs from 16 Austronesian-speaking populations in Taiwan and the Philippines. Ross et al.^36^ examined 700 variants of a single folktale across 31 countries in Europe. Smith et al.^42^ computed *F*_ST_ values of public goods game contributions between Hadza camps. Additionally, Muthukrishna et al.^38^ also calculated pairwise cultural *F*_ST_ values based on the World Value Survey between the US and other countries, as well as between China and other countries. They found between-country *F*_ST_ values ranging from 0.10 to 0.20, and within-country *F*_ST_ values of up to 0.12. With the exception of the estimate in Bowles^44^ which has been critiqued as inflated^43^, estimates of genetic variation between populations (lower panel of Fig. 2) are considerably lower. Notably, in a recent analysis of 27 adjacent ethnic groups from the Kasai Central Province of the DRC, the largest genetic *F*_ST_ observed was 0.002^45^.

### Cultural differentiation predicts cooperation

**Fig. 3:**
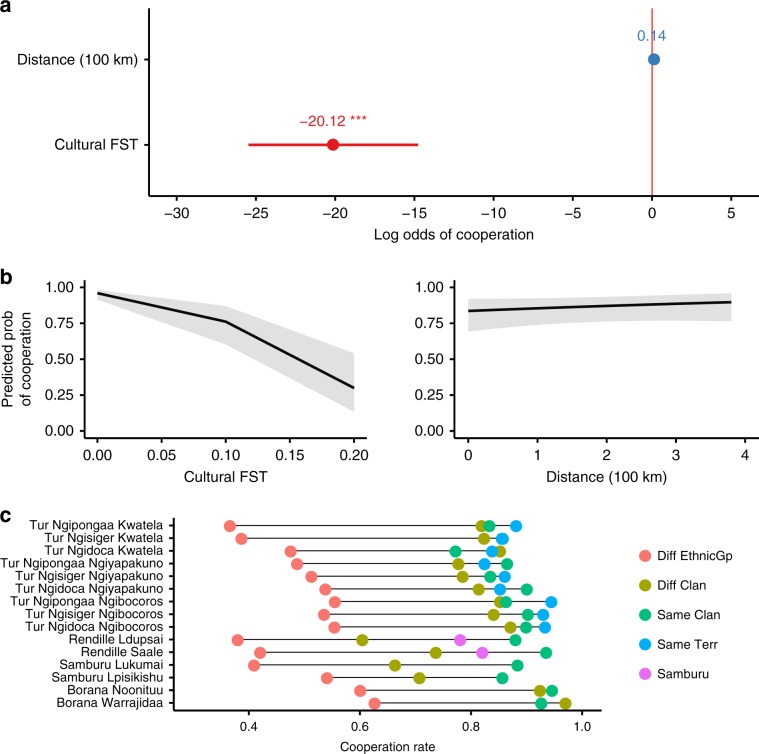
Predictors of cooperation rates between groups. Logistic regression model of the effect of cultural *F*_ST_ and geographic distance between actor and target’s group, on whether subject endorses the cooperative act. **a** Log-odds estimates with 95% CI (***p < .001). **b** Predicted probabilities of subjects endorsing cooperative act conditioned on cultural *F*_ST_ value and geographic distance between actor and target’s group. **c** Average rate of endorsement of cooperative act by vignette condition for each subpopulation. For the Turkana (Tur) there are a minimum of 12 subjects in each vignette condition per clan per territorial section. For the Rendille, Samburu and Borana there are a minimum of 17 subjects per clan in each vignette condition. Source data are provided as a Source Data file.

The population structure of cultural variation explains the prevalence of norms of cooperation among unknown individuals, confirming a key prediction of CGS theory. Cultural *F*_*ST*_ has a significant negative effect (Log Odds = −20.12, *p* *<* .001) on the prevalence of norms requiring people to cooperate with unknown individuals from another group (Fig. 3 and Supplementary Table 3). The magnitude of the effect is substantial. For instance, an increase in cultural *F*_ST_ value from 0.05 to 0.15 between actor and target’s group nearly halves the predicted probability of a subject endorsing the cooperative act (Fig. 3b). Geographic distance does not have a significant effect on the prevalence of cooperative norms (Log Odds = 0.14, *p* *<* .1). The pseudo *R* squared of the model was computed using the theoretical method of the *r.squaredGLMM* function in the MuMin package in *R*. The marginal *R* squared (variance explained by the fixed effects) is 0.18, and the conditional R squared value (variance explained by the fixed plus random effects) is 0.57.

### Warfare norms have strongest effect on cooperation

The population structure of norms governing raiding has the strongest effect on the social scale of cooperation. Since we used norms from five categories of pastoral life to measure cultural differentiation—cooperation, crime and punishment, raiding, cultural markers, and family dynamics—we additionally computed *F*_ST_ values between pairs of groups using only norms from each category. When using these five *F*_ST_ subcategories as predictors in the mixed effect logistic regression model, *F*_ST_ values based on raiding norms and on cultural marker norms are the only predictors that have a significant effect (Supplementary Fig. 5). The effect size of *F*_ST_ based on raiding (Log-Odds = −19.28) is considerably larger than that of cultural markers (Log-Odds = −6.02). This suggests that norms governing warfare are most strongly influenced by CGS. Although it is not a prediction we could make a priori because of uncertainty about which norms impact group success, the observation bolsters the interpretation that between group competition generates the observed correspondence between population structure and cooperation.

### Correlation of geographic distance and cultural differentiation

*F*_ST_ and geographic distance are positively correlated (Pearson’s correlation coefficient = 0.93, *p* < .001) (Supplementary Fig. 6). Geographic distance is most correlated with *F*_ST_ of norms pertaining to cultural markers, and to cooperation (0.9), and is least correlated with norms pertaining to raiding (0.63). Since *F*_ST_ of raiding norms is also the strongest predictor of cooperation, the association between cooperation and *F*_ST_ is unlikely to be due to geographic distance. Rather, our causal interpretation of the associations is that geographic distance leads to cultural differentiation, which leads to CGS. CGS facilitates cooperative norms at the scale of cultural differentiation. Warfare is a high-stakes form of cooperation to acquire crucial limiting resources that get shared widely within communities^6,39^. Therefore, norms governing conduct of warriors on the battlefield is under particularly strong selection via CGS in this ethnographic context.

### Cooperation rates by subpopulation

The effect of cultural *F*_ST_ and geographic distance on cooperation shows the same trend in all of the 15 subpopulations we studied (Supplementary Figs. 7–8). The Borana have the highest cooperation rates overall, particularly when the target is from a different ethnic group (Fig. 3c). The Borana sampled, coming from the Waso region of northern Kenya, are strongly influenced by Islam, where a more generalized ethos of giving is emphasized. This alludes to the possibility that CGS may be operating on scales larger than what we have measured here, such as religious groups, which may partly explain cooperative norms extending across ethnic boundaries^16,18,46^. Consistent with ethnographic impressions, the Rendille are atypically cooperative with the Samburu (Fig. 3c). Subjects from both Rendille clans endorsed cooperation with the Samburu at higher rates than they endorse cooperation with another Rendille clan, despite being more culturally similar to other Rendille. Being smaller and militarily weak, the Rendille depend on maintaining a friendly relationship with the larger, militarily stronger Samburu. This suggests that other processes of norm evolution, such as deliberation among elders, may have shaped norms specifying how Rendille interact with the Samburu^47^.

### Cooperation rates by vignette scenario

The same association of cultural *F*_ST_ and geographic distance on cooperation is seen across all the vignette scenarios (Supplementary Figs. 9–10). The two vignette scenarios that elicited the highest cooperation and the least decline as *F*_ST_ increased involved sharing relief food with a poor and hungry woman, and aiding an injured man heading to a hospital for treatment. The targets are in particularly vulnerable situations and likely to elicit the most empathy. Alternately, because they pertain to services offered by the nation state, it may be that norms governing these actions derive from the process of nation state building, and therefore extend beyond ethnic boundaries. The scenario with the lowest levels of cooperation involved entrusting a stranger to sell one’s cow in town with the expectation that this person will bring the money to the owner afterwards. Such a level of trust likely requires the additional scaffolding of repeated interactions, suggesting that CGS may have greater scope to influence norms regulating transient interactions. Rates of cooperation were not significantly different between vignette scenarios that entailed not harming a target versus scenarios that entailed helping a target (Supplementary Table 4).

## Discussion

In conjunction with the findings reported in refs. ^15,48^, our results lend credence to the idea that cultural group selection facilitated the evolution of large-scale cooperation in humans. The correspondence we have found between the population structure of cultural variation and the social scale of cooperative norms confirms a key untested prediction of the CGS theory. A landmark study showed that there is sufficient cultural differentiation between countries for CGS to be plausible^15^, but the study did not assess patterns of cooperation to show that it coincided with the patterning of cultural differentiation. The most direct support for CGS to date is that increased competition between economic firms caused cooperation within firms to increase^48^. While that study confirmed a predicted relationship between competition and cooperation, it did not assess cultural variation and cooperation at different social scales in nested populations. In addition, it is unclear to what extent patterns of differentiation and competition between nation states and firms generalize to the kinds of societies that humans have lived in for most of our evolutionary history. The top-down formal regulations that influence cultural variation and competition in modern nation states and firms differ from the bottom-up informal processes at work in decentralized tribal-scale societies. The acephalous populations in our study compete intensively for livestock, grazing territory, and water resources, and participate in intergroup raids that affect resource availability and population growth. Such a competitive regime may be more akin to how groups interacted for much of human history^49–52^. Thus, our results imply that CGS could potentially have sculpted the human cooperative psychology, and not just contemporary norms.

Competing evolutionary theories of cooperation cannot easily account for the patterns we document, without incorporating CGS. One theory is that humans’ anomalous cooperative laxity comes from the misfiring of a psychology that evolved in a social milieu in which people knew each other well^24–27^. Because geographic proximity would be a basis for familiarity, the lack of evidence for geographic distance influencing cooperative norms is hard to fit with this account. Another possibility is that people are more willing to cooperate with culturally similar individuals because they can coordinate more easily with people who play by the same rules^28,32^. However, while in coordination games there is no incentive to defect as long as your partner cooperates, in the scenarios of cooperation our subjects responded to, individuals help or resist taking from others. Thus, individuals give up some benefit in order to engage in the cooperative act, and social sanctions would be needed to achieve norm compliance. A third possibility is that the content of norms is shaped by within-group evolutionary processes arising from individuals practicing and enforcing norms from which they stand to benefit^29–31,33–35,53^. In this account, norms sustaining cooperation are ones that individuals recognize to be beneficial, or that are beneficial to certain individuals in positions of influence, like leaders or rulers. However, this account cannot readily explain the social scale at which we observe cooperation. The territories of the Turkana, Samburu, Borana and Rendille regularly shift and overlap. As such, the ecological benefits of sharing pasturelands with another clan would likely extend to sharing with clans of another cultural population. Moreover, there are evident gains to be had from the emergence of norms that deter cattle raiding of other cultural populations. In the Kwatela area of Turkana, 20% of male mortality (50% of adult male mortality) is due to cattle raiding^39^. Individual herders are acutely aware of these costs, and elders periodically attempt to negotiate peace treaties. Yet the prevalent norms promote cattle raiding of other ethnic groups, while also deterring cattle raiding against nearly a million other Turkana. Similar raiding norms prevail in the other populations we studied (Supplementary Table 2 and Supplementary Fig. 7 RAID and STEALTH scenarios). While CGS can account for this puzzle, within-group norm evolution processes that do not incorporate CGS do not. Lastly, it is possible that CGS acting over a long period of human evolutionary history could have led to the genetic evolution of predispositions to cooperate with unknown individuals^3,4,7,12,14^. If so, then individuals would preferentially create and adopt cooperative norms that benefit the cultural group, causing within-group norm evolution to produce norms that benefit the cultural group. Our data cannot parse out to what extent these norms are the direct result of CGS, or the result of a within-group norm evolutionary processes stemming from a psychology shaped by CGS. However, if the latter were a dominant force, it should accelerate CGS by generating between-group cultural differences in norms impacting group success.

We conclude that group-level selection on cultural variation has likely left a mark on the human cooperative psychology and continues to influence which social norms and institutions prevail in human societies. This could explain why despite humans’ unprecedented cooperative scope, we are nonetheless culturally parochial. Furthermore, if CGS has been ongoing in human evolutionary history then it could have shaped other social phenomena such as violence, morality, and religion, as some authors have suggested^6,18^. Finally, if CGS is influencing contemporary norm shifts, it is a useful framework for modeling social change in multi-cultural nation states by linking the processes of immigration, acculturation, and extension of social support. While research spanning many disciplines is needed to uncover the micro and macro processes underlying cultural change, the way forward might be smoother if the dynamics of cultural group selection are considered.

## Methods

### Social organization of study populations

We sampled 793 individuals from 9 clans (2 clans each from Borana, Rendille and Samburu, and 3 clans from Turkana), as well as 3 Turkana territorial sections (Table 1 and Fig. 1). The ethnic groups in the study—Borana, Rendille, Samburu and Turkana—are all organized around patrilineal descent based clans and sub clans, detailed in Supplementary Figures 1–4. Among the Samburu and Rendille, men frequently settle close to their fathers’ home areas when establishing an independent household, and settlements tend to be clan-specific. Borana and Turkana clans are more geographically dispersed although concentrations of one clan may be found in certain locations. In the case of the Turkana, clans cross-cut the territorial sections, so a particular clan can be found in multiple territorial sections. Women adopt the clan, territorial section, and ethnic identity of their husbands if they are officially married (marriage in which bride price has been paid); otherwise they retain their father’s group identities.

### Survey development and piloting process

We drew on our prior ethnographic field experience in these societies (CH has worked among the Rendille, Borana, and Samburu for 13 years, and SM among the Turkana for 10 years) to design questionnaires that would be comprehensible and salient to all the study populations.

To create the survey to measure cultural differentiation, we produced an initial list of 170 normative beliefs that spanned a broad range of social contexts that would be pertinent across the entire study population: crime and punishment, family dynamics, cooperation and helping, cultural markers, and theft and raiding. To create the vignette scenarios to measure cooperation, we initially developed 26 scenarios that encompassed a range of familiar contexts in which people would regularly cooperate. To generate these initial norms and vignettes, we complemented our existing ethnographic knowledge with exploratory field interviews conducted in a settlement of the Ngiyapakuno territorial section of the Turkana, where SM has maintained a longterm semi-permanent field station. To ensure comprehension and salience across all four ethnolinguistic groups, we focused on norms and vignettes that we expected would be contextually relevant to arid pastoralists in Kenya more generally.

We piloted the original questions in one location of the Ngiyapakuno territorial section of the Turkana. During this pilot phase we pruned down the normative questionnaire from 170 to 49 norms, and the vignette scenarios from 26 to 16 so that a subject could complete the study in about 1 to 1.5 h. The decisions on which norms to include were based on further assessments regarding ease of participant comprehension, variety of domains, and contextual salience. Supplementary Tables 1 and 2 display the final list of traits and vignettes we used in the study. By limiting the pilot stage to one location of one territorial section of one ethnic group, we minimized the risk of selecting norms that would bias our questionnaire in a way that would increase the *F*_ST_ estimates between clans, territorial sections, or ethnic groups, as majority of the pairwise population comparisons would be among populations who were not involved in the pilot study.

The norms were phrased as statements that participants could either agree or disagree with. Wording within the statements was varied between affirmative and negative declarations to avoid biased verbal agreement. All surveys were translated into the local languages by local research assistants who were fluent in their native language and competent in English. The translations were refined over an iterative back-translation and re-translation process to ensure the word choice conveyed analogous concepts to members of the four ethnic groups.

### Choice of data collection sites

Aside from the Ngiyapakuno and Kwatela territorial sections of the Turkana, the clans and territorial sections included in the study were determined after the pilot phase was completed and the questionnaire content was finalized. This reduced bias and specificity towards any particular clan when designing the survey instrument. We also needed to be flexible in locating large concentrations of clan members opportunistically, depending on their current migration and settlement patterns. The two Turkana territorial sections that had been pre-selected, the Ngikwatela and the Ngiyapakuno, have previously participated in studies undertaken from SM’s research camp, which is located in their areas, and were therefore easily accessible populations. Once in the field, and taking into consideration settlement densities, security, and ease of access, we selected: the Ngibochoros territorial section as the third Turkana territorial section; the Ngisiger, Ngipongaa, and Ngidoca for Turkana clans; the Lukumai of Lkisin and Lpisikishu of Naisunyai for the Samburu clans; the Warrajidaa and Noonituu Borana clans of Kinna; and the Saale and Ldupsai Rendille clans of Namarei. See Fig. 1 for the locations of these populations.

### Data collection

CH conducted 3 field trips spanning 8 months between April 2015 and July 2016 to pilot the questionnaires, collect data, and to train local field assistants who would continue to collect data. During data collection, research teams consulted each household within a circumscribed location to assess the ethnic and clan identities of each available family member. Effort was made to balance gender and ages within the sample, and only one member of each household was interviewed in order to capture individual variation within populations. The data collection process was digitized using *Open Data Kit* on the *ONA* platform, so that questionnaires could be administered and data entered by local field research assistants using hand-held tablets. Typically, interviews lasted from 45 min to 1.5 h. A total of 759 individuals completed the questionnaire pertaining to cultural beliefs. All but four of these participants also completed the 16 vignette scenarios. In addition, 34 Rendille subjects responded only to the vignette scenarios with “Samburu” specified as the different ethnic group target.

### Calculating cultural differentiation

Using the number of subjects who agree with the normative statement, we computed the frequency of each of the 49 norms in each of the clans, territorial sections and ethnic groups. We then calculated a pairwise cultural *F*_ST_ value for each norm and an average pairwise *F*_ST_ value across all 49 norms. The pairwise calculations were done for each pairing of the four ethnolinguistic groups and for each pairing of the clans within each ethnolinguistic group. For the Turkana, we also paired each of the 3 Turkana territorial sections. The *F*_ST_ value of trait *x* between populations *i*,*j* is the ratio of between-group variance to the total variance in trait *x*, and is given by:

$$F_{{\mathrm{ST}}\,x} = \frac{{\frac{{n_i}}{{n_i + n_j}}(p_i - \bar p)^2 + \frac{{n_j}}{{n_i + n_j}}(p_j - \bar p)^2}}{{\bar p\left( {1 - \bar p} \right)}}$$

where *n*_*i*_ and *n*_*j*_ are the number of individuals sampled from populations *i* and *j*, *p*_*i*_ and *p*_*j*_ are the frequency of trait *x* in populations *i* and *j*, and *p̄* is the overall freqeuncy of trait *x* in populations *i* and *j*. The numerator is the variance in the frequency of trait *x* between populations *i* and *j* weighted by the sample size of populations *i* and *j*; the denominator is the total variance in the trait *x*. When *F*_ST_ values are 0, all the variation in the trait is between individuals within a group and is not structured between populations, leaving no scope for CGS. Conversely, when *F*_ST_ values are 1, all of the variation in the trait is structured between groups, and CGS will dominate the evolutionary dynamics.

Although *F*_ST_ values of norms impacting group success should have the greatest impact on CGS, we used a range of norms pertaining to pastoral livelihood to obtain robust estimates of the scale of cultural differentiation. The scale at which social learning occurs influences the acquisition of a wide range of norms, and so norms that do not influence group success will co-occur with norms that do. Moreoever, norms that do not obviously have to do with cooperation or group success may, unknowing to its practitioners and scholars, have large impacts on group success. Of the 49 norms, 10 pertained to cooperation, 9 to crime and punishment, 9 to raiding, 10 to family dynamics, and 11 were norms that we (subjectively) judged to be cultural markers, conventions that may identify individuals as members of a particular group.

### Measurement of cooperation rates

To assess the social scale of cooperative norms, we used 16 vignette scenarios (Supplementary Table 2) in which the main character is in a position to affect the well-being of the target character. In 12 of the scenarios, the main character could help or refuse to help the target in some way (for e.g., by sharing or refusing to share a water well). In the remaining 4 scenarios, the main character could harm or not harm the target in some way (for e.g., by stealing or not stealing their livestock). This allowed us to incorporate culturally relevant situations in which refraining from harming is the mode by which people cooperate. We systematically varied the scenarios so that the target, who was always unknown to the main character, was either from the same clan, from a different unspecified clan of the same ethnolinguistic group, or from a different unspecified neighboring ethnolinguistic group as the main character. In the case of the Turkana, we had additional conditions in which the target was from the same or different territorial section as the main character. The Rendille maintain an atypically friendly relationship with the Samburu and refrain from raiding them^47^. So, for the Rendille, we had a fourth classification in which the target was identified as a Samburu. Each participant was assigned one condition (e.g., different clan) and responded to each of the 16 vignettes saying whether what the primary character did to the target was right or wrong. The frequency of endorsement of the cooperative action (helping, or not harming) gives the prevalence of cooperative norms across the social boundary specified by the vignette condition. Note that although we measure both cultural differentiation and the scale of cooperation by measuring normative beliefs and practices, they measure distinct things. Whereas cultural differentiation tracks whether people adhere to a certain cultural norm, the scale of cooperation specifies to whom norms regarding helping apply.

### Measurement of geographic distance

We accounted for geographic distance between populations because it could influence levels of cooperation, and covary with cultural differentiation. In particular, there is greater scope for people to know individuals from communities located near them. Marriage, market exchange, and overlap in use of grazing areas and watering sites could generate interpersonal ties between individuals of nearby communities. Reputational considerations could cause people to cooperate with individuals who come from these communities. Because CGS could be operating along with these reputational considerations, we expect that increasing the cultural *F*_ST_ will decrease cooperation between communities when geographic distance is held constant. We recorded the GPS location of subjects and computed the geographic distance from each subject of one community to all subjects from the other community. The average of these distances is our measure of geographic distance between pairs of groups.

### Regression analysis

To assess if the scale of cultural variation influences the scale of cooperation, we performed a mixed-effect logistic regression using the *glmer* function of the lme4 package in R. The dependent variable is whether a subject endorses the cooperative act towards the target. Subject ID, vignette scenario, and the subject’s lowest level group membership (e.g., Samburu Lpisikishu, or Turkana Ngiyapakuno Ngidoca) are included as random effects. The fixed effects are the cultural *F*_ST_ value and the geographic distance between the actor’s and target’s social groups (e.g., the cultural *F*_ST_ value and geographic distance between two Samburu clans for a Samburu subject assigned to the “different clan” vignette condition). The *F*_ST_ values assigned to a subject are averages of the pairwise *F*_ST_ values of all pairs that represent the relation between actor (who is from subject’s population), and the target who is specified by vignette condition. Thus, for a Samburu subject assigned to the “different clan” vignette condition the *F*_ST_ value assigned is the pairwise *F*_ST_ value between the two Samburu clans we sampled. For a Turkana subject assigned to the “different clan” condition, because three clans were sampled, it is the average of three pairwise *F*_ST_ values between each of the three Turkana clans. For subjects assigned to the “different ethnic group” condition, it is the average of six pairwise *F*_ST_ values between each of the four ethnic groups we sampled. For the Rendille subjects in the “Samburu” vignette condition, it is the pairwise *F*_ST_ value between the Rendille and Samburu. Geographic distance was similarly assigned.

### Research ethics

The study was done in compliance with all relevant ethical regulations. Informed consent was obtained from each participant after the nature and possible consequences of the studies were explained. The protocol was approved by the Institutional Review Board of Arizona State University.

### Reporting summary

Further information on experimental design is available in the Nature Research Reporting Summary linked to this paper.

## Supplementary information

Supplementary Information

Reporting Summary

## Data Availability

The data that support the findings of this study is provided as a Source Data file and can be accessed from the Open Science Framework using the link 10.17605/OSF.IO/HRJK7.

## Source data

Source Data

## Code availability

The R code used to analyze the data and produce Fig. 3, Supplementary Figs. 5–10, and Supplementary Tables 3–4 is available as a Source Code file and can be accessed from the Open Science Framework with the identifier 10.17605/OSF.IO/HRJK7. The analyses were conducted using R version 3.6.0.
