# Supplementary material for: Human large-scale cooperation as a product of competition between cultural groups
Source: Nat Commun. 2020 Feb 4;11:702. doi: 10.1038/s41467-020-14416-8 (PMC7000669; doi:10.1038/s41467-020-14416-8)
Supplement: Supplementary file 1 — Supplementary Information [file 41467_2020_14416_MOESM1_ESM.pdf]

# Supplementary Information

## Human large-scale cooperation as a product of competition between cultural groups.

Handley and Mathew

# 1. Supplementary Figures

**Supplementary Figure 1:** Borana social organization. Marriage is exogamous at the moiety level, and individuals live in settlements where clans are intermixed. *Clans included in this study.

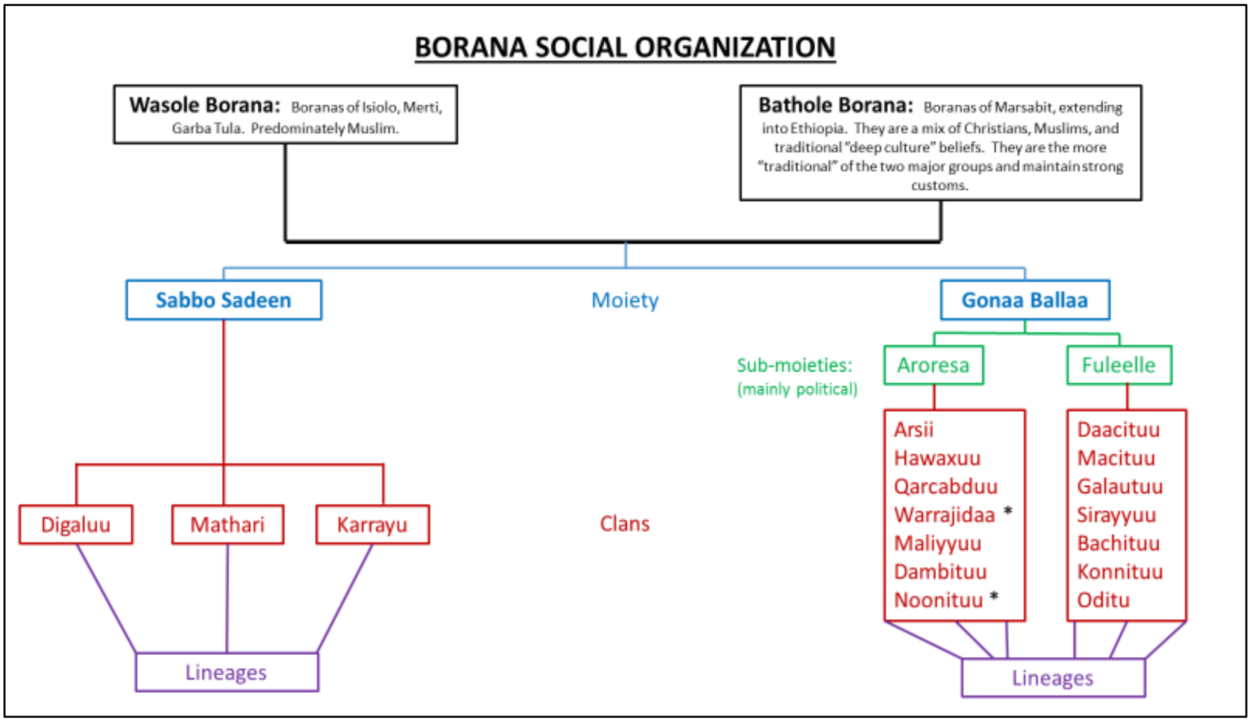

**Supplementary Figure 2:** Rendille social organization. Marriage is exogamous at the clan level, and clans can marry within their own phratry. Individuals live in settlements comprised only of their own clan members. *Clans included in this study.

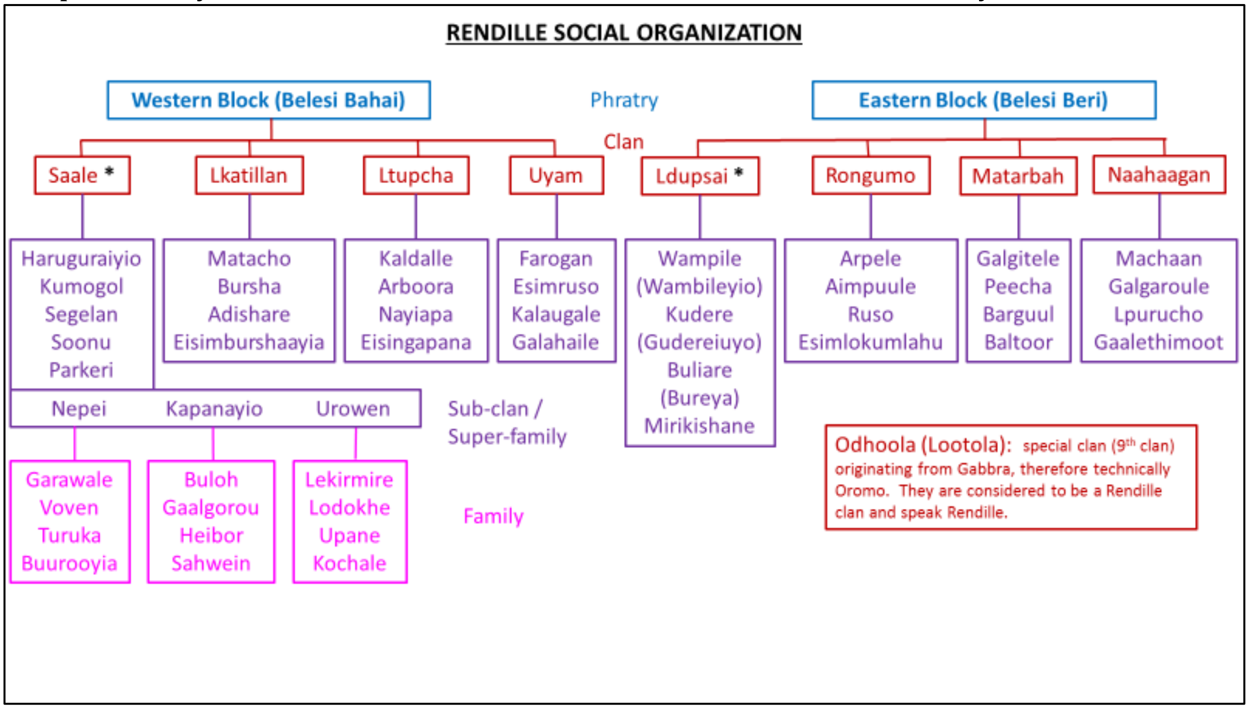


**Supplementary Figure 3:** Samburu clan organization. Marriage is exogamous at the clan level, and clans can marry within their own phratry. Individuals live in settlements comprised only of their own clan members. *Clans included in this study.

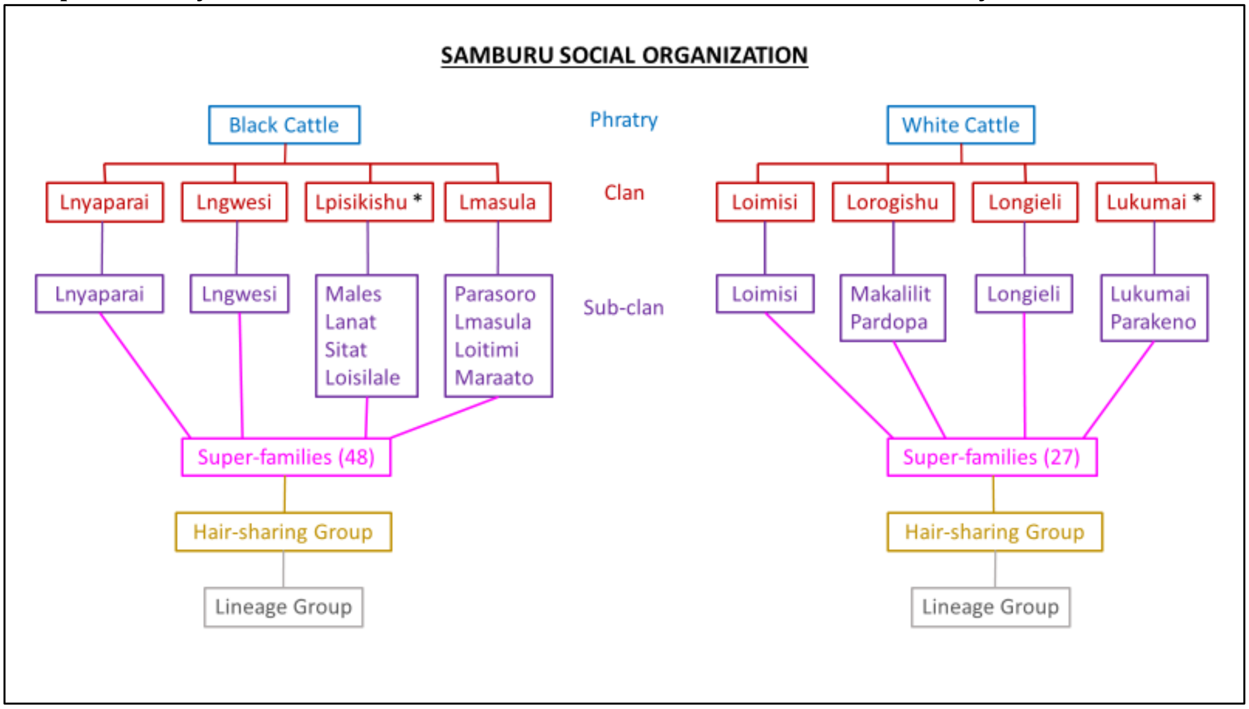

**Supplementary Figure 4:** Turkana clan organization. Marriage is exogamous at the clan level, and clans can marry within their own territorial section. Individuals live in settlements comprised only of member from their own territorial section but with the clans intermixed. *The three territorial sections and three clans that were included in this study.

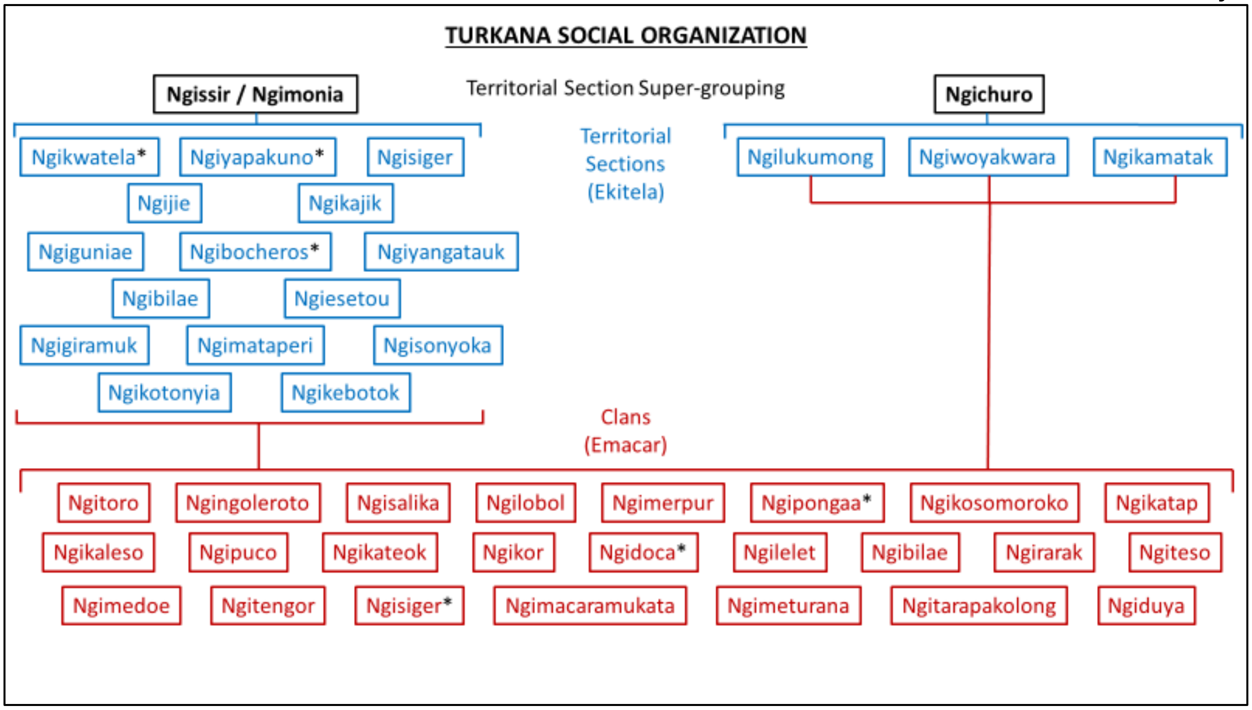

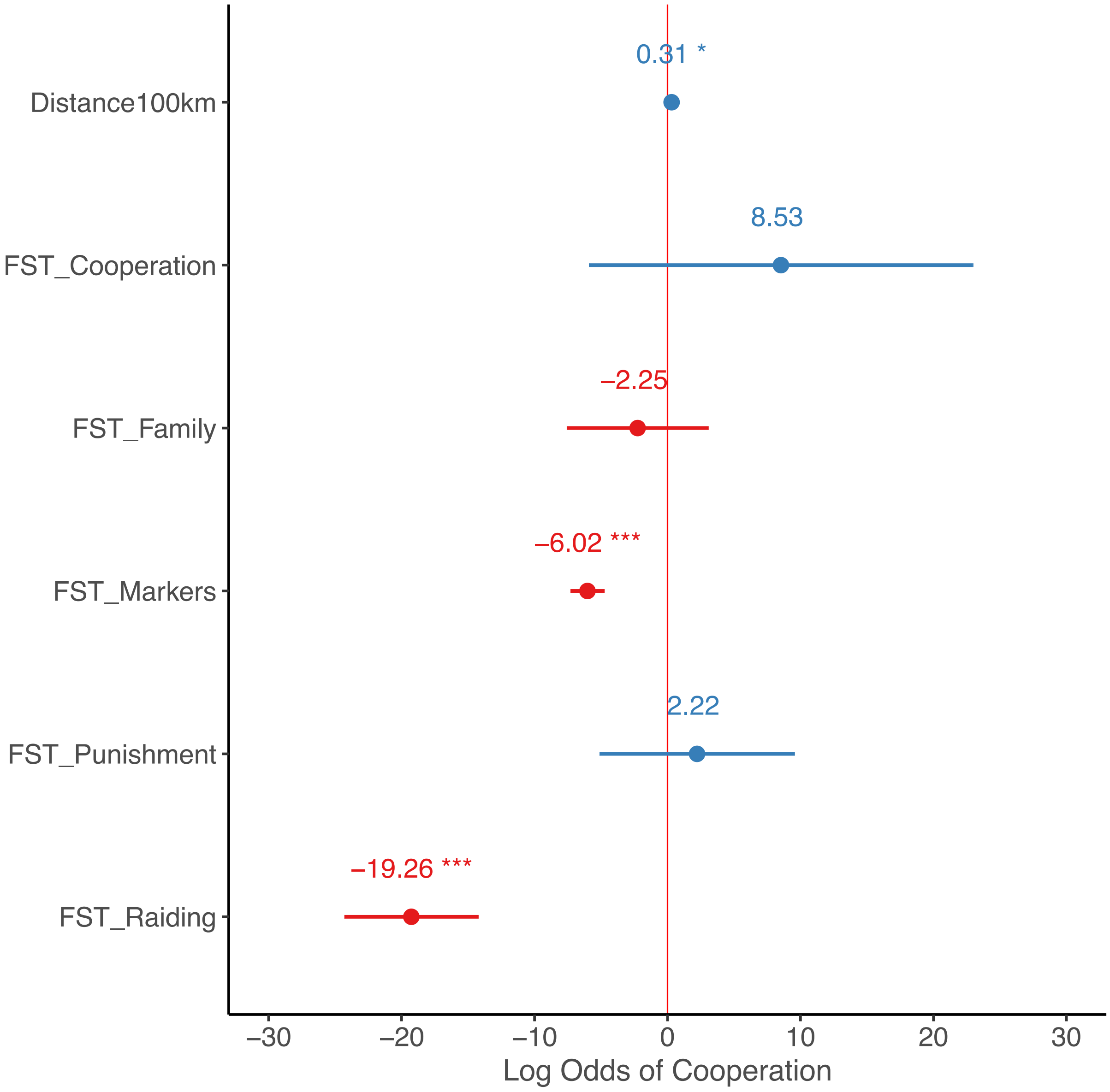


**Supplementary Figure 5:** Log Odds of endorsing the cooperative act from a logistic regression model with FST values based on norm categories, and geographic distance as predictors. Subject ID, subpopulation and vignette scenario are included as random effects. *** indicates p<.001, and * indicates p<.05. Error bars show 95% confidence interval.

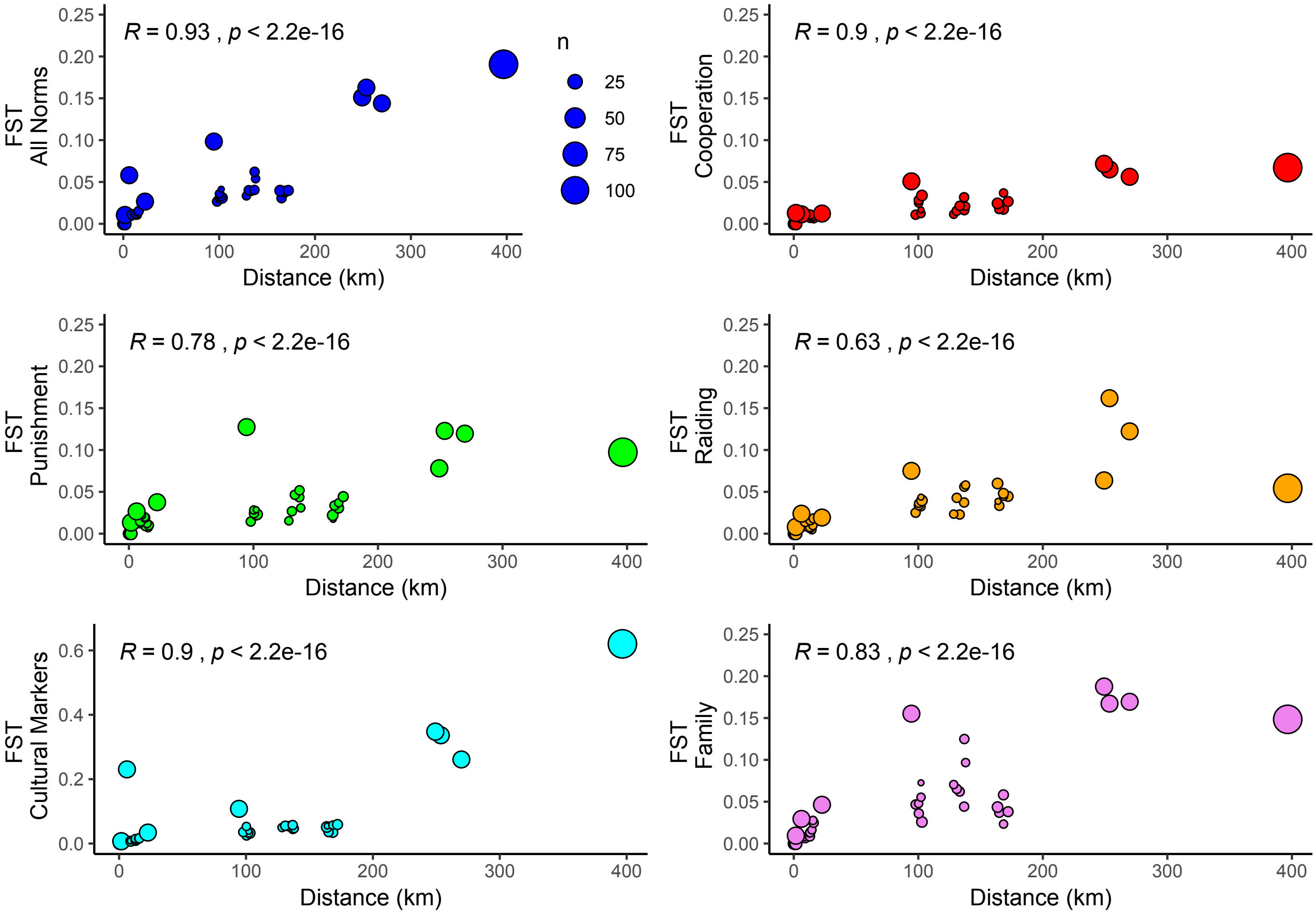


**Supplementary Figure 6:** Correlation between the predictors of cooperation rates in the logisitic regression model. Subplots show correlation between geographic distance and FST values using all norms, and FST values based on subcategories of norms. Each circle represents a population of subjects assigned to a particular vignette condition (e.g. Borana-Different Ethnic Group Condition, or, Lukumai-Different Clan Condition.) Size of the circles (see legend of first subplot) shows the number of subjects in that subpopulation-vignette condition pairing. The largest circle represents multiple clans and territorial sections of the Turkana who were assigned to the “Different Ethnic Group Condition”. Correlation coefficients printed on each plot are estimated using Pearson’s method.

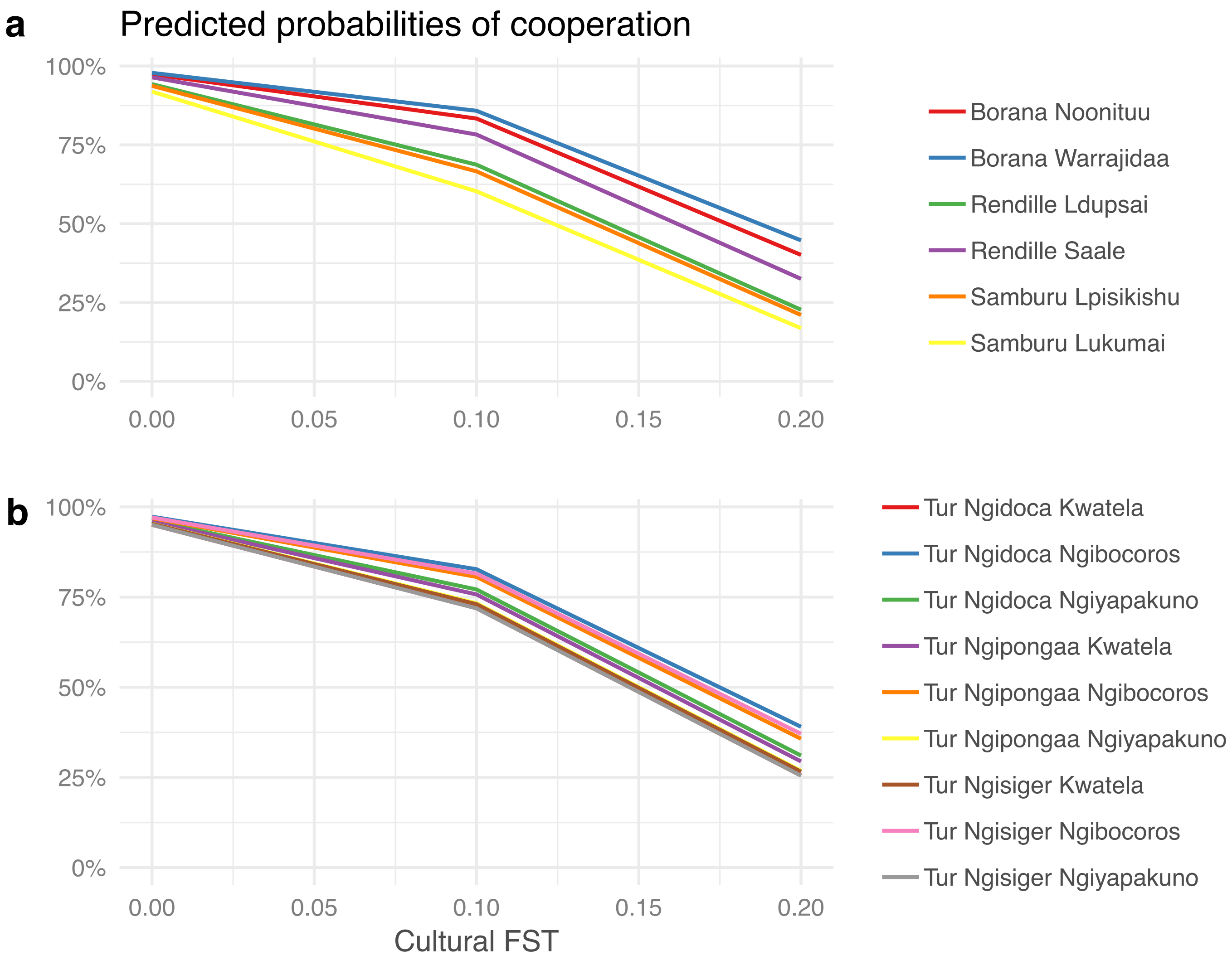


**Supplementary Figure 7:** Predicted probabilities of subjects endorsing the cooperative act conditioned on cultural FST values between the actor and target's group, for each of the 15 subpopulations studied. For visual clarity, 8 of the subpopulations are shown in top panel, and the other 8 are shown in the bottom panel.

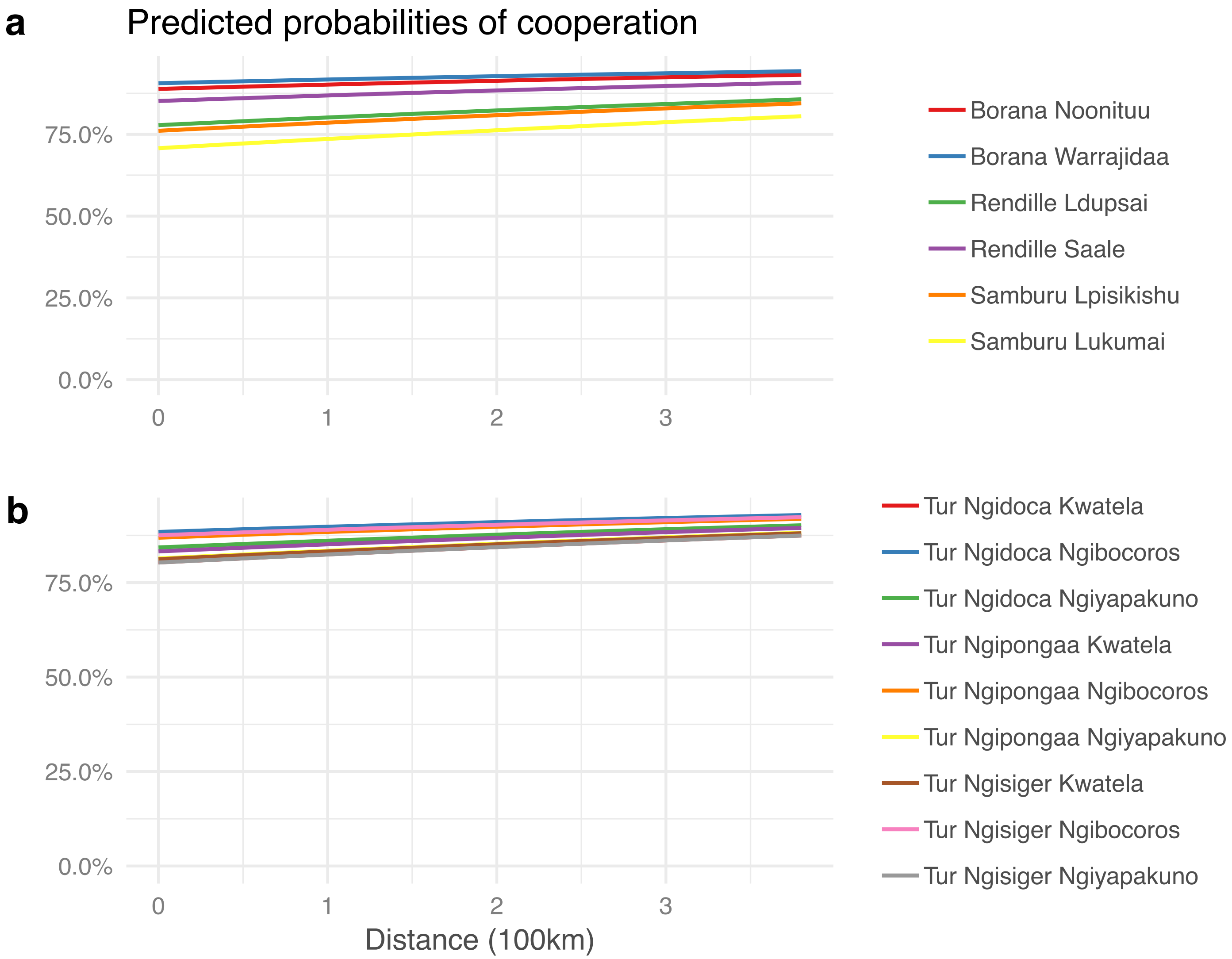


**Supplementary Figure 8:** Predicted probabilities of subjects endorsing the cooperative act conditioned on geographic distance between the actor and target's group, for each of the 15 subpopulations studied. For visual clarity, 8 of the subpopulations are shown in top panel, and the other 8 are shown in the bottom panel.

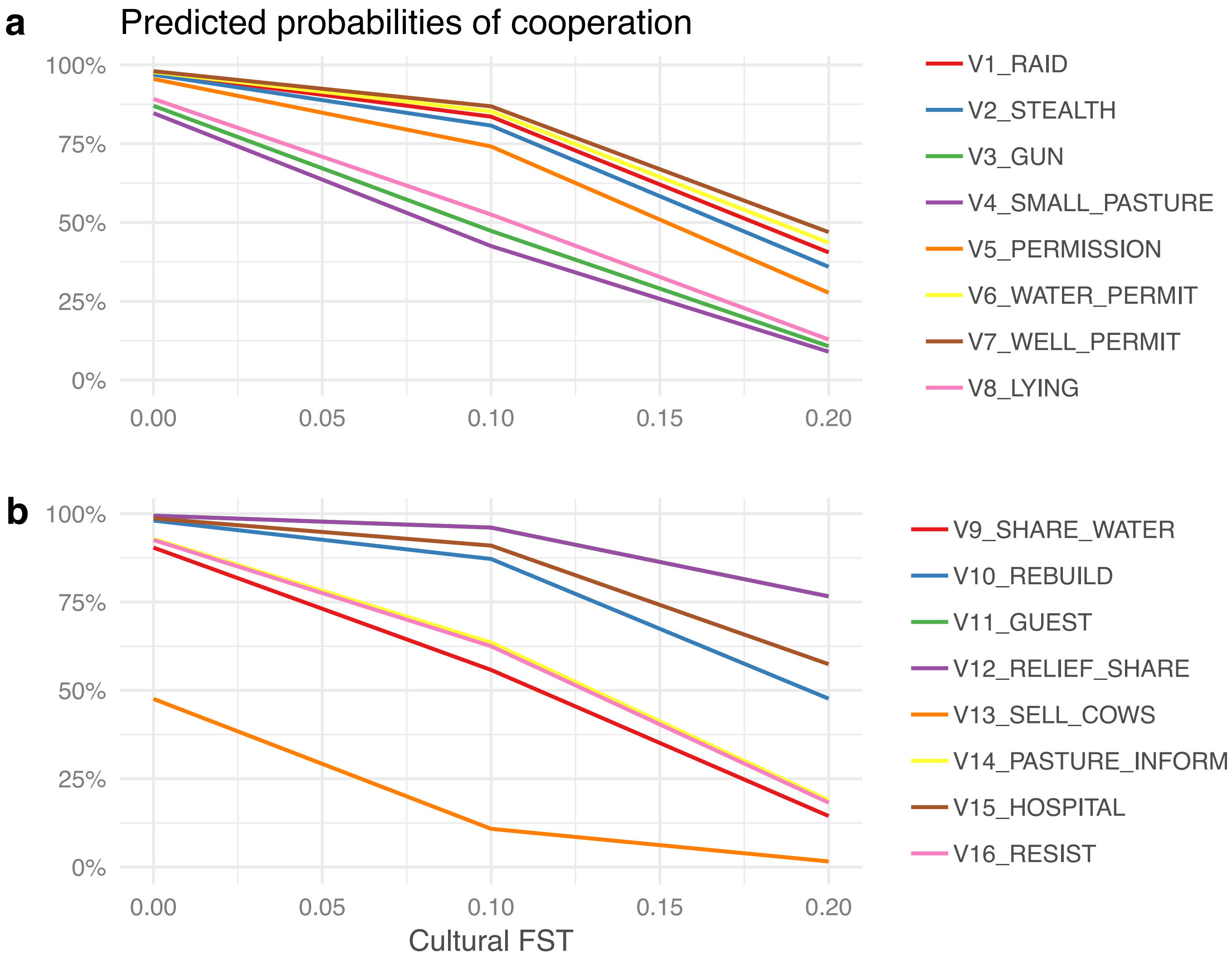


**Supplementary Figure 9:** Predicted probabilities of subjects endorsing the cooperative act conditioned on cultural FST values between the actor and target's group, for each of the 16 vignette scenarios used to assess cooperation. For visual clarity, 8 of the scenarios are shown in top panel, and the other 8 are shown in the bottom panel.

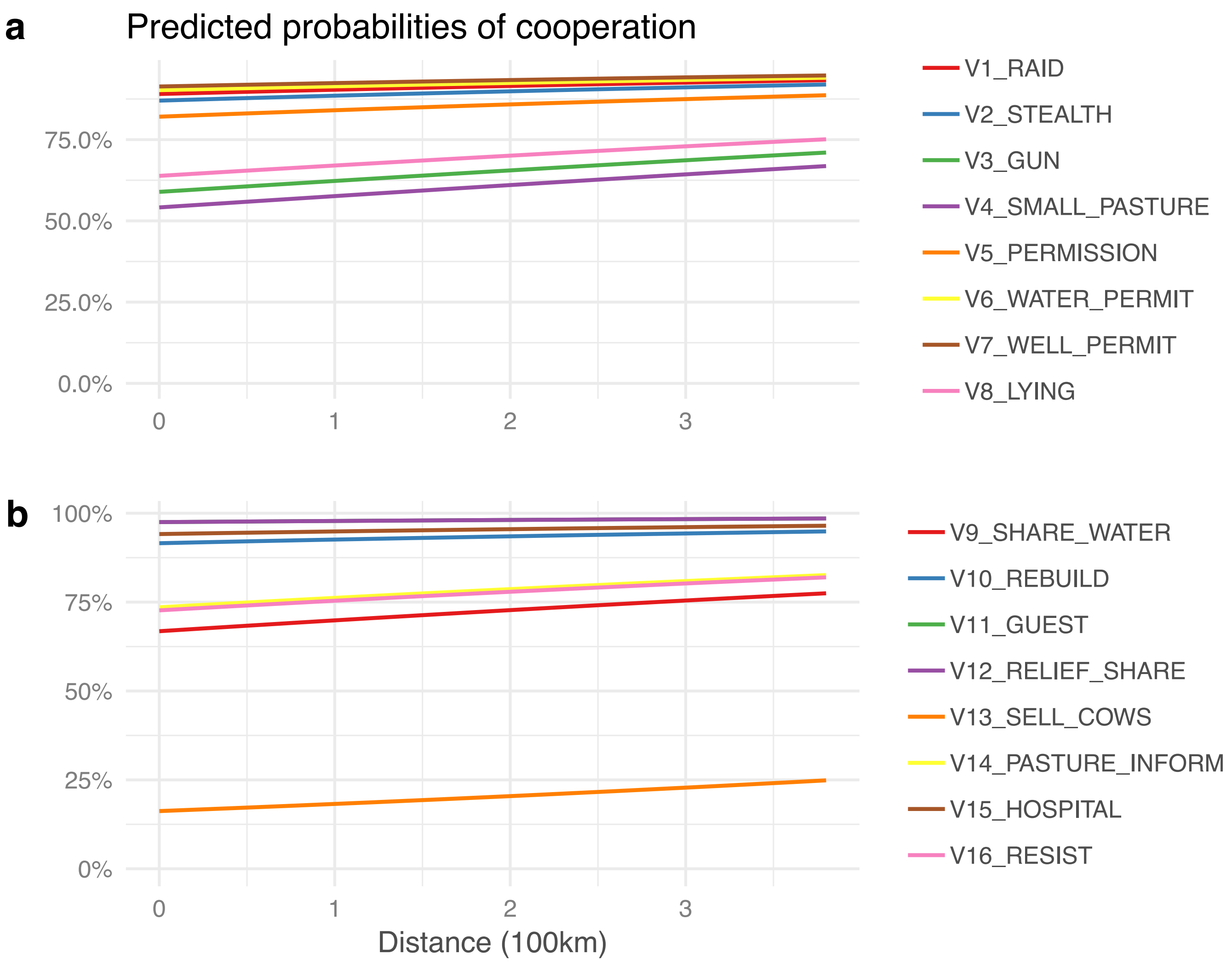


**Supplementary Figure 10:** Predicted probabilities of subjects endorsing the cooperative act conditioned on geographic distance between the actor and target's group, for each of the 16 vignette scenarios used to assess cooperation. For visual clarity, 8 of the scenarios are shown in the top panel, and the remaining 8 are shown in the bottom panel.

## Supplementary Tables

**Supplementary Table 1:** Normative statements used to measure cultural differentiation between populations. The norms are ordered here according to their category. The order in which the norms were presented to the subjects is shown in the codebook of the Source Data file. All subjects had the same ordering.

| Norm Label | Description | Category |
|---|---|---|
| 1. FIND MARK | When you are leaving your home area to find new grazing, it is necessary to find an area in which there are many people of your clan. | cooperation |
| 2. INFO GRAZE | When grazing in your area is good, you must share the information first with others from your clan before others from a different clan. | cooperation |
| 3. RESCUE | On a raid, a warrior needs to protect and rescue only his close companions, not the men he does not know. | cooperation |
| 4. SELFISH | It is okay to not share meat with a selfish man. | cooperation |
| 5. VISITOR SLAUGHTER | When a friend comes from far to visit you, you must slaughter an animal for him. | cooperation |
| 6. HOST | When you are traveling to an area where you don't know anyone, it is necessary to find someone of your clan to host you. | cooperation |
| 7. POOR WOMAN | If a woman is poor, it is okay if she does not share her maize flour with others who ask. | cooperation |
| 8. REFUSE POOR | When a relative who lost his animals travels from far to ask you for an animal, you can refuse to assist him if your animals are few. | cooperation |
| 9. STRANGER AGEMATE | A person should help an age-mate who is unfamiliar to him from a far-away place the same way he would help an age-mate that he knows from his own place. | cooperation |
| 10. WELL | If somebody you don't know of your clan asks to use your well, you must agree. | cooperation |
| 11. BEAT AGEMATES | It is good for age-mates to gather and beat a fellow age-mate who is behaving badly. | crime & punishment |
| 12. NEIGHBOR CHILD | If a woman sees her neighbor's child misbehaving, it is okay for her to cane this child. | crime & punishment |
| 13. RECONCIL-IATION | After a person is beaten by his age-mates, there must be something done to reconcile him back to the group. | crime & punishment |
| 14. VIOLATE PEACE | Anybody who violates a peace agreement by stealing should be punished. | crime & punishment |
| 15. AGEMATE NO REPORT | A man should be disciplined if he witnesses an age-mate committing a wrong-doing and does not report this to others. | crime & punishment |
| 16. EXCOMMUN-ICATED | A person can be excommunicated from (your ethnic) society if he does something very evil. | crime & punishment |
| 17. HUNGER STEAL | A young boy can steal a goat from the neighbor's herd if he is hungry. | crime & punishment |
| 18. MURDER KILL | If a person murders someone, the victim's family can kill one member of the killer's family. | crime & punishment |

| | | |
|---|---|---|
| 19. COWARD | A warrior who behaves like a coward on a raid should be punished by his age-mates. | crime & punishment |
| 20. COWARD SHAME | If an individual from one age-group behaves like a coward on a raid, it brings shame to the entire age-group. | raiding |
| 21. KIDNAP | It is permitted to kidnap women or children of the enemy during a raid. | raiding |
| 22. KILL WOMEN | It is permitted to kill women in the course of a raid. | raiding |
| 23. RAID INNOCENT | It is permitted to raid from a neighboring tribe even if they don't raid (your ethnic group). | raiding |
| 24. RAIDING BLESSINGS | A raiding party should not depart if they have not received blessings from the elders or a diviner. | raiding |
| 25. UNMARRIED RAID | Only unmarried men should go on raids. | raiding |
| 26. WOUNDED | During a raid, if the fight is tough, you can leave a wounded man to be killed by the enemy. | raiding |
| 27. RAID PRESSURE | If a warrior's age-mates are going on a raid, then he must also go. | raiding |
| 28. SONG | It is good for a warrior to boast about the raids he has gone on through his bull song. | raiding |
| 29. CIRCUMCISED | Men should be circumcised at the time of initiation. | cultural markers |
| 30. DONKEY | It is okay to eat the meat of a donkey. | cultural markers |
| 31. FIRE | A woman should never let her fire die out. | cultural markers |
| 32. INTESTINES | Intestines should be eaten only by women and uninitiated men | cultural markers |
| 33. JOKE | A woman can only joke with a man from her husband's age-set. | cultural markers |
| 34. LADY FOOD | A young lady cannot eat in front of young men unless it is a family member. | cultural markers |
| 35. SHAVE | When someone dies, some family members shave the hair on their forehead. | cultural markers |
| 36. SHEEP | It is okay to drink the milk of sheep. | cultural markers |
| 37. SICK | When the animals are sick, one goat is killed and its chyme is spattered on the other animals. | cultural markers |
| 38. SKIN TUCK | A married woman should not tuck her skin under her when sitting. | cultural markers |
| 39. UMBILICAL | Male babies' umbilical cords are cut using a spear. | cultural markers |
| 40. ADOPTION | It is good to give a child to a grandparent or another relative for adoption. | family dynamics |
| 41. EXTENDED | Brideprice must be given not only to the parents of the girl, but also to her extended family. | family dynamics |
| 42. FATHERS AREA | When married, a man should stay within his father's area. | family dynamics |
| 43. HUSBAND RAPE | A husband can have sex with his wife whenever he wants, even if she refuses. | family dynamics |

| | | |
|---|---|---|
| 44. LOVING FATHER | A good father should carry and play with his children and show them affection. | family dynamics |
| 45. MOTHER CARE | Even after a daughter is married and leaves home, her mother should look out for her and take care of her needs. | family dynamics |
| 46. SCOLD | A woman can scold her husband if he has done something wrong. | family dynamics |
| 47. MANYATTA INITIATION | When an age-set is established, the families of the boys should construct a manyatta and live together for several months. | family dynamics |
| 48. SUICIDE | It is acceptable for a person to commit suicide if they lose all of their animals in a drought. | family dynamics |
| 49. MANYATTA JUNIORS | Junior elders must come together with their age mates to a manyatta to learn about how to behave like elders. | family dynamics |

**Supplementary Table 2:** Vignettes scenarios to assess cooperation. The personal names used in the vignettes were changed to reflect common names found within each ethnic group. The vignettes were classified into those where the central character could harm or avoid harming the target, and ones in which the central character could help or refuse to help the target. Subjects are considered to endorse cooperation if they endorse the action of not harming, or the action of helping the target. The vignettes are listed here in the order in which they were presented to subjects. All subjects had the same ordering.

| Vignette Label | Description | Classification |
|---|---|---|
| 1. RAID | Mamalo and his agemates' animals have reduced in number so they decide to organize a raid. They launch an attack against Rendilles of their same clan / different clan / different ethnic group. What is the relationship between Mamalo and the people who were raided? Do you think what Malmalo did was right or wrong? | Don't Harm/ Harm |
| 2. STEALTH | Mamalo and a few of his friends decide that they will steal some goats to eat. They go and steal from a family of their own clan / different clan / different ethnic group. Mamalo and his friends manage to grab two goats while the owners are sleeping, and no one was hurt in the incident. They roast the meat and are happy. What is the relationship between Mamalo and the family who had animals taken? Do you think what Mamalo and his friends did was right or wrong? | Don't Harm/Harm |
| 3. GUN | During the drought, Gisewa has moved with his animals into a border area. While watering his animals at a communal water point, a man confronts him to say that Gisewa should not have watered his animals before him. This man belongs to Gisewa's clan / different clan / different ethnic group. Gisewa and the man escalate to fighting one another. Gisewa fires some shots at the man to make him run away. What is the relationship between Gisewa and the man? Do you think it was right or wrong of Gisewa to use his gun to scare away the man? | Don't Harm/ Harm |
| 4. SMALL PASTURE | While travelling on a long journey, Koni passes through a small, uninhabited area with excellent grazing. He sees that it could support a small number of animals for some time but probably not large numbers of animals. He decides that he wants to bring his animals back here for pasture. The next day, he encounters a man searching for pasture with | Help/Don't Help |

| | | | |
|---|---|---|---|
| | | his animals. The man belongs to Koni's own clan / different clan / different ethnic group. Koni knows that the man would benefit from taking his animals to the grazing area that he saw. What is the relationship between Koni and the man? Must Koni tell the man about the grazing area? | |
| 5. | PERMIS-SION | Leringa is an elder in his community located along a boundary area. During a particularly dry period, a number of families have travelled to his area in search of pasture. The families were from Leringa's own clan / different clan / different ethnic group. They asked Leringa whether they would be able to graze their animals within his area, even though they know that the area is very dry. What is the relationship between Leringa and the families searching for pasture? Is it a must that Leringa allow them to graze in his area? | Help/Don't Help |
| 6. | WATER PERMIT | Goyipi lives in a boundary area and manages the use of the local borehole. A number of new families have moved close to the area, as their own borehole has become dry. The families are from Goyipi's own clan / different clan / different ethnic group. They ask Goyipi whether they may use the borehole to water their animals, as it is the closest borehole to them. The borehole is already supporting many animals. What is the relationship between Goyipi and the families that moved into the area? Must Goyipi allow the families to use the borehole? | Help/Don't Help |
| 7. | WELL PERMIT | Algayo lives in a boundary area and has dug a very deep well for his animals, as the current conditions are very dry. A new family has moved into the area close to where Algayo is living. The new family is from Algayo's own clan / different clan / different ethnic group. As the family is limited by not having any young men to dig a well for them, they ask whether or not they may use Algayo's well to water their animals while they are living in his area. What is the relationship between Algayo and the family that has moved into his area? Must Algayo give the family permission to use the well that he dug? | Help/Don't Help |
| 8. | LYING | Malmalo is herding his animals in a border area and comes across a stray goat. There is no one around, and Malmalo decides to eat the goat. Late that evening, the owner of the goat comes looking for it and asks Malmalo if he has seen the goat. The man belongs to the same clan / different clan / different ethnic group as Malmalo. Malmalo says that he has not seen the goat. Do you think it was right or wrong of Malmalo to not tell the man that he ate his goat? | Harm/Don't Harm |
| 9. | SHARE WATER | As Koni is walking along the road on, he crosses paths with a man who is on a long journey. The man belongs to the same clan / different clan / different ethnic group as Koni. The man notices that Koni has a small bottle of water for his trip and asks Koni for his water. Koni has far to go, and it is only a small bottle of water. What is the relationship between Koni and the man? Must Koni give his water to the man? | Help/Don't Help |
| 10. | REBUILD | Derache lives in a boundary area and has only a small number of animals in his herd. During a time of peace, a man comes to him to ask for an animal to rebuild all those that he has lost due to drought. Derache knows that the man has worked hard to keep his animals, but he was unfortunate that his animals died. The man belongs to the same clan / different clan / different ethnic group as Derache. What is the relationship between Derache and the man looking for animals? Must Derache give the man an animal? | Help/Don't Help |
| 11. | GUEST | A woman is travelling through Ariyo's area on a long journey. The woman asks Ariyo if she can spend the night in her home and be given some food for her journey. The woman belongs to Ariyo's own clan / different clan / | Help/Don't Help |

| | | |
|---|---|---|
| | different ethnic group. What is the relationship between the woman and Ariyo? Must Ariyo provide the woman with a place to sleep and something to eat? | |
| 12. RELIEF SHARE | Wareya has just collected her relief food when she encounters a woman who came late and missed the distribution. The woman has travelled far to get to the distribution. She belongs to the same clan / different clan / different ethnic group as Wareya. The woman asks Wareya for some of her relief food. What is the relationship between Wareya and the woman? Must Wareya give the woman some of her relief food? | Help/Don't Help |
| 13. SELL COW | Malmalo is on his way to the cattle market to sell a cow. Suddenly, he becomes very sick and realizes that he cannot continue on his journey, but he must sell his cow. There is another man on the road going to the same market as Malmalo. The man says that he can take Malmalo's cow to the market for him to sell and promises to return with the money to Malmalo. The man belongs to the same clan / different clan / different ethnic group as Malmalo. What is the relationship between Malmalo and the man? Can Malmalo trust the man to sell his cow and bring him back the money? | Help/Don't Help |
| 14. PASTURE INFORM | While travelling on a long journey, Koni passes through an area with plenty of water and grazing available. The next day, he encounters a herder searching for pasture with his animals. The herder belongs to Koni's own clan / different clan / different ethnic group. What is the relationship between Koni and the herder? Must Koni tell the herder about the plentiful pasture and water that he saw? | Help/Don't Help |
| 15. HOSPITA L | Derache is traveling far from home and encounters a wounded man who is struggling to walk. Derache talks to him and finds that the man belongs to his own clan / different clan / different ethnic group. The wounded man asks Derache if he can carry him to the hospital. What is the relationship between Derache and the wounded man? Must Derache help to carry the wounded man to hospital? | Help/Don't Help |
| 16. RESIST | One day as Algayo was on a long journey from his home area, he saw that a settlement was under attack. The settlement was of people belonging to his own clan / different clan / different ethnic group. Algayo could see that it was a tough fight and the people were losing their animals. Must Algayo join the men as they resist the raiders? | Help/Don't Help |

**Supplementary Table 3:** Logistic Regression Model Results

Generalized linear mixed model fit by maximum likelihood (Laplace Approximation) ['glmerMod']

Family: binomial ( logit )

Formula: Help ~ FSTVal + Distance100km + (1 | SubID) + (1 | VigScenario) + (1 | RandomEffectGp)
Data: data_long

| AIC | BIC | logLik | deviance | df.resid |
|---|---|---|---|---|
| 9774.9 | 9819.6 | -4881.5 | 9762.9 | 12631 |

**Scaled residuals:**

| Min | 1Q | Median | 3Q | Max |
|---|---|---|---|---|
| -8.5850 | -0.1006 | 0.2101 | 0.4036 | 6.0648 |

**Random effects:**

| Groups | Name | Variance | Std.Dev. |
|---|---|---|---|
| SubID | (Intercept) | 0.8686 | 0.9320 |
| VigScenario | (Intercept) | 1.9434 | 1.3941 |
| RandomEffectGp | (Intercept) | 0.1861 | 0.4314 |

Number of obs: 12637, groups: SubID, 793; VigScenario, 16; RandomEffectGp, 15

**Fixed effects:**

| | Estimate | Std. Error | z value | Pr(>\|z\|) |
|---|---|---|---|---|
| (Intercept) | 2.9829 | 0.3733 | 7.990 | 1.35e-15 *** |
| FSTVal | -20.1185 | 2.5574 | -7.867 | 3.64e-15 *** |
| Distance100km | 0.1410 | 0.1336 | 1.055 | 0.291 |

Signif. codes: 0 '***' 0.001 '**' 0.01 '*' 0.05 '.' 0.1 ' ' 1

**Correlation of Fixed Effects:**

| | (Intr) | FSTVal |
|---|---|---|
| FSTVal | 0.009 | |
| Distnc100km | -0.047 | -0.969 |

**Supplementary Table 4:** Logistic Regression comparing “Harm/Don’t Harm” versus “Help/ Don’t Help” Vignette Scenarios

*(Variable labeled “HarmVignette” is a dummy variable coded as 1 if vignette scenario is a “Harm/Don’t Harm” scenario, and 0 if it is a “Help/Don’t Help” vignette scenario.)*

```
Generalized linear mixed model fit by maximum likelihood (Laplace Approximation)
['glmerMod']
 Family: binomial  ( logit )
```

---

```
Formula: Help ~ FSTVal + Distance100km + HarmVignette + (1 | SubID) +     (1 |
VigScenario) + (1 | RandomEffectGp)
   Data: data_long
```

---

```
    AIC      BIC   logLik deviance df.resid
 9776.7   9828.8  -4881.3   9762.7    12630
```

---

```
Scaled residuals:
    Min      1Q  Median      3Q     Max
-8.5900 -0.1006  0.2103  0.4034  6.0626
```

---

```
Random effects:
 Groups         Name        Variance Std.Dev.
 SubID          (Intercept) 0.8685   0.9320
 VigScenario    (Intercept) 1.9088   1.3816
 RandomEffectGp (Intercept) 0.1861   0.4314
Number of obs: 12637, groups:  SubID, 793; VigScenario, 16; RandomEffectGp, 15
```

---

```
Fixed effects:
              Estimate Std. Error z value Pr(>|z|)
(Intercept)     3.0911     0.4209   7.344 2.08e-13 ***
FSTVal        -20.1185     2.5637  -7.847 4.25e-15 ***
Distance100km   0.1410     0.1339   1.053    0.292
HarmVignette   -0.4325     0.7986  -0.542    0.588
```

---

```
Signif. codes:  0 ‘***’ 0.001 ‘**’ 0.01 ‘*’ 0.05 ‘.’ 0.1 ‘ ’ 1
```

---
