# Supplementary material for: Human large-scale cooperation as a product of competition between cultural groups
Source: Nat Commun. 2020 Feb 4;11:702. doi: 10.1038/s41467-020-14416-8 (PMC7000669; doi:10.1038/s41467-020-14416-8)
Supplement: Supplementary file 2 — Reporting Summary [file 41467_2020_14416_MOESM2_ESM.pdf]

nature research

Corresponding author(s): Sarah Mathew

Last updated by author(s): Dec 21, 2019

Reporting Summary

Nature Research wishes to improve the reproducibility of the work that we publish. This form provides structure for consistency and transparency in reporting. For further information on Nature Research policies, see Authors & Referees and the Editorial Policy Checklist.

Statistics

For all statistical analyses, confirm that the following items are present in the figure legend, table legend, main text, or Methods section.

n/a	Confirmed	
☐	☒	The exact sample size (*n*) for each experimental group/condition, given as a discrete number and unit of measurement
☐	☒	A statement on whether measurements were taken from distinct samples or whether the same sample was measured repeatedly
☐	☒	The statistical test(s) used AND whether they are one- or two-sided<br>*Only common tests should be described solely by name; describe more complex techniques in the Methods section.*
☐	☒	A description of all covariates tested
☒	☐	A description of any assumptions or corrections, such as tests of normality and adjustment for multiple comparisons
☐	☒	A full description of the statistical parameters including central tendency (e.g. means) or other basic estimates (e.g. regression coefficient) AND variation (e.g. standard deviation) or associated estimates of uncertainty (e.g. confidence intervals)
☐	☒	For null hypothesis testing, the test statistic (e.g. *F*, *t*, *r*) with confidence intervals, effect sizes, degrees of freedom and *P* value noted<br>*Give P values as exact values whenever suitable.*
☒	☐	For Bayesian analysis, information on the choice of priors and Markov chain Monte Carlo settings
☐	☒	For hierarchical and complex designs, identification of the appropriate level for tests and full reporting of outcomes
☐	☒	Estimates of effect sizes (e.g. Cohen's *d*, Pearson's *r*), indicating how they were calculated

Our web collection on statistics for biologists contains articles on many of the points above.

Software and code

Policy information about availability of computer code

Data collection	Data collection was done using Ona, Open Data Kit, and Microsoft Excel.
Data analysis	Data analysis was done using R version 3.6.0, and Microsoft Excel

For manuscripts utilizing custom algorithms or software that are central to the research but not yet described in published literature, software must be made available to editors/reviewers. We strongly encourage code deposition in a community repository (e.g. GitHub). See the Nature Research guidelines for submitting code & software for further information.

Data

Policy information about availability of data

All manuscripts must include a data availability statement. This statement should provide the following information, where applicable:

- Accession codes, unique identifiers, or web links for publicly available datasets
- A list of figures that have associated raw data
- A description of any restrictions on data availability

The data that support the findings of this study is provided as a Source Data file and can be accessed from the Open Science Framework with the identifier DOI 10.17605/OSF.IO/HRJK7

Field-specific reporting

Please select the one below that is the best fit for your research. If you are not sure, read the appropriate sections before making your selection.

☐ Life sciences ☒ Behavioural & social sciences ☐ Ecological, evolutionary & environmental sciences

For a reference copy of the document with all sections, see nature.com/documents/nr-reporting-summary-flat.pdf

Behavioural & social sciences study design

All studies must disclose on these points even when the disclosure is negative.

Study description	Quantitative data was obtained using survey questionnaires to assess normative beliefs, and vignette scenarios in which subjects were assigned to one of multiple conditions.
Research sample	793 adult pastoralists from northwest Kenya from the Borana, Rendille, Samburu and Turkana ethnic groups. The proportion of female subjects in the sample was 63%. We sampled individuals from the Warrajidaa and Noonituu clans of the Borana, the Ldupsai and Saale clans of the Rendille, the Lukumai and Lpisikishu clans of the Samburu, and the Ngipongaa, Ngidoca and Ngisiger clans from the Ngibocoros, Ngiyapakuno and Ngikwatela territorial sections of the Turkana. Sample is representative of individuals within these clans.
Sampling strategy	Convenience sampling procedure was used. During data collection, research assistants consulted each household within a circumscribed location to assess the ethnic and clan identities of each available family member. Effort was made to balance gender and obtain a wide age range of adults within the sample. Only one member of each household was interviewed. Sample size was predetermined based on an assessment of how much time it would take to find and complete the questionnaire with subjects, and how much resources we could allocate for implementing the study.
Data collection	Surveys were designed through an ONA platform and accessed via the ODK application on hand-held Samsung tablets. Typically, interviews lasted from 45 minutes to 1.5 hours. Other individuals in the household were present for some of the interviews. While all interviews were administered by a single research assistant, a researcher or another research assistant were sometimes also present.
Timing	Data was collected between April 2015 and July 2016.
Data exclusions	No data were excluded from the analysis.
Non-participation	4 of the 793 participants did not complete the study.
Randomization	Participants were allocated to the vignette conditions at random.

Reporting for specific materials, systems and methods

We require information from authors about some types of materials, experimental systems and methods used in many studies. Here, indicate whether each material, system or method listed is relevant to your study. If you are not sure if a list item applies to your research, read the appropriate section before selecting a response.

Materials & experimental systems

n/a	Involved in the study	
☒	☐	Antibodies
☒	☐	Eukaryotic cell lines
☒	☐	Palaeontology
☒	☐	Animals and other organisms
☐	☒	Human research participants
☒	☐	Clinical data

Methods

n/a	Involved in the study	
☒	☐	ChIP-seq
☒	☐	Flow cytometry
☒	☐	MRI-based neuroimaging

Human research participants

Policy information about studies involving human research participants

Population characteristics	See above
Recruitment	Participants were recruited by going household to household in the areas that were identified as having members of the particular clans being sampled. The sample is biased towards individuals who are within the home during the daytime. As a result, we had more females in the sample than males.
Ethics oversight	Institutional Review Board of Arizona State University

Note that full information on the approval of the study protocol must also be provided in the manuscript.
